# Sensitivity and specificity of spinal cord Magnetic Resonance Imaging in the diagnosis of HTLV-1 associated myelopathy

**DOI:** 10.1186/1742-4690-11-S1-P12

**Published:** 2014-01-07

**Authors:** Luiz CF Romanelli, Rafael HC Bastos, Luciana C Silva, Tatiana Martins, Débora B Reiss, Gabriela S Freitas, Mariana A Souza, Cláudia LF Horiguchi, João GR Ribas, Aline M Silva, Fernando A Proietti, Marina L Martins, Anísia da SD Ferreira, Anna Bárbara FC Proietti

**Affiliations:** 1 Faculdade da Saúde e Ecologia Humana (FASEH), Vespasiano, Minas Gerais, Brazil; 2 Medicina Diagnóstica ECOAR, Belo Horizonte, Minas Gerais, Brazil; 3 Grupo Interdisciplinar de Pesquisa em HTLV (GIPH), Belo Horizonte, Minas Gerais, Brazil

## 

Since 1997, the Interdisciplinary Research Group on HTLV-1/2 (GIPH) has been following individuals infected with HTLV in Minas Gerais, Brazil, in an open prospective cohort study. The HAM diagnosis is based on clinical parameters, with support of complementary exams to exclude other possible causes of myelopathy. The objective of this study was to evaluate the sensitivity and specificity of spinal cord MRI in HAM diagnosis. This was a cross-sectional study, conducted between March 17^th^ and September 28^th^, 2012. The 120 HTLV-1 seropositive were included in the study. HAM was diagnosed on the basis of the World Health Organization diagnostic criteria. Cervical and thoracic MRIs were performed in sagittal and axial sequences fast spin-echo T2-weighted, without gadolinium. Blinded interpretation of MRIs was performed by radiologists who did not know clinical neurologic status of the participants. The mean age of the 120 individuals was 48.0 ± 12.8 years (range: 16.4 to 72.6 years) and 77 (64.2%) were women. Among all patients, 19 (15.8%) had a diagnosis of HAM. There was no statistically significant difference between the mean ages of the HAM and asymptomatic groups (p = 0.7). In the HAM group, 7 (36.8%) of them showed changes in MRIs compatible with the disease. On the other hand, 93 (83.0%) asymptomatic individuals have had normal MRIs. Spinal cord MRI demonstrated to be a diagnostic method with good specificity but low sensitivity in the diagnosis of HAM. It is possible that time evolution of disease, as well as the degree of neurological impairment is directly related to the sensitivity of the method.

